# Corneal biomechanical properties are associated with the activity and prognosis of Angioid Streaks

**DOI:** 10.1038/s41598-018-26430-4

**Published:** 2018-05-25

**Authors:** Shotaro Asano, Kosuke Nakajima, Kana Kure, Keiko Azuma, Kimiko Shimizu, Hiroshi Murata, Tatsuya Inoue, Ryo Obata, Ryo Asaoka

**Affiliations:** Department of Ophthalmology, Graduate School of Medicine and Faculty of Medicine, The University of Tokyo, Tokyo, 113-8655 Japan

## Abstract

The aim of the current study is to investigate corneal biomechanical properties in detail using Ocular Response Analyzer (ORA) and Corvis ST (CST) tonometry and to analyze the association between corneal biomechanical properties and the frequency of intravitreal anti-vascular endothelial growth factor (VEGF) injections (F_IV_) in AS eyes with choroidal neovascularization (CNV). Twenty-eight eyes of 15 patients with AS were enrolled. Mean age of AS patients was 67.9 ± 9.8 years. ORA and CST measurements were carried out, in addition to comprehensive ophthalmic examinations. LogMAR visual acuity (VA) and ΔVA (the change of VA from baseline to the final visit) were calculated in each eye. Also, the relationships between F_IV_, and the variables of initial age at the observation period, axial length, and corneal biomechanical properties were investigated in eyes with AS using linear mixed model with model selection using AICc. In 28 AS eyes, 16 eyes underwent intravitreal anti-VEGF injections during follow-up period. Lower corneal hysteresis (CH), higher corneal resistant factor (CRF) and higher CST measured the DA ratio were associated with the increase of F_IV_ in AS eyes (p = 0.01, p = 0.002, p = 0.027, respectively), suggesting the usefulness for monitoring of corneal biomechanical properties.

## Introduction

Angioid streaks (AS) is known as a type of collagen disease^1^, which represents the dehiscence of elastic lamina of the Bruch’s membrane^2^. The diagnosis of AS is typically made through funduscopic examination and fluorescein angiography (FA). Although the exact mechanism of the onset of AS has remained unclear^3^, AS appears to develop associated with the forces distributed to Bruch’s membrane^4^, including intrinsic or extrinsic mechanical stress related to ocular muscle traction^1^, trauma, and pressure on the eyes^5^. The ingrowth of fibrovascular tissue and subsequent break of brittle Bruch’s membrane take place in AS^6^, and as a result, eyes with AS have a high risk of the development of secondary choroidal neovascularization (CNV)^3^. CNV is a major cause of visual loss in AS, and, as observed with optical coherent tomography (OCT) and OCT angiography, subretinal hemorrhage and/or subretinal fluid appear once CNV emerges^2,7^. When CNV develops, eyes are usually treated with intravitreal anti-vascular endothelial growth factor (VEGF) injection^8^. In turn, the disease activity of AS is reflected on the frequency of the intravitreal anti-VEGF injection (F_IV_)^2^.

It is currently possible to measure corneal biomechanical properties, such as hysteresis, using Ocular Response Analyzer (ORA; Reichert Ophthalmic Instruments, Buffalo, New York, USA). A previous study has indicated that the ORA measured-corneal resistance factor (CRF) was increased in AS, compared with age-matched controls^3^. Another study reported that corneal hysteresis (CH) and CRF were both decreased in eyes with a connective tissue disorder of Marfan’s syndrome^9^. Recently, the detailed assessment of different aspects of corneal biomechanical properties, such as stiffness and elasticity, have become possible, with the development of Corvis ST (CST; Oculus, Wetzlar, Germany)^10^. A detailed evaluation of corneal biomechanical properties with CST may be useful in investigating the progression of AS. We therefore measured the corneal biomechanical properties in detail in eyes with AS using ORA and CST, and the relationship between F_IV_ and corneal biomechanical parameters were analyzed. Moreover, we investigated the relationship between ORA- and CST-measured corneal biomechanical properties and visual outcomes.

## Methods

### Study Population

In the current study, we retrospectively reviewed the charts of patients in the outpatient clinic of the University of Tokyo Hospital. The current study was in accordance with the tenets of the Declaration of Helsinki and was approved by the institutional Review Board (IRB) of the University of Tokyo as a retrospective review of the patients’ medical records. All data were fully anonymized before we accessed them. Informed consent was obtained to participate the current research, and participants who did not grant authorization to use their medical records for the research were excluded from analyses.

Participants were recruited at the outpatient clinic of the University of Tokyo Hospital, Department of Ophthalmology. The inclusion criteria were: 1) diagnosis of AS with a funduscopic examination and FA; the characteristic clinical appearance included peripapillary ring which irregularly radiates linear streaks^2^; and, 2) a follow-up interval longer than one year. Exclusion criteria were: 1) a history of ocular disease other than AS; 2) intraocular surgery, including cataract, during the follow-up period; 3) pathological ocular surfaces condition, which may affect corneal biomechanical property measurements and, 4) refractive errors of more than ±2.00 diopters. The eyes in the control group were 1) those who did not have any known ophthalmologic abnormalities on slit-lamp anterior segment examination and funduscopic examinations, 2) no intraocular surgery, including cataract, during the follow-up period, 3) no pathological ocular surfaces condition, which may affect corneal biomechanical property measurements, and 4) without refractive errors of more than ±2.00 diopters.

### Follow-up examinations and treatment protocol for AS

All patients underwent comprehensive ophthalmic examinations including the measurement of best-corrected visual acuity (BCVA), slit-lamp biomicroscopy, funduscopy, and OCT examinations at each visit. The BCVA was measured as a decimal VA using the Landort C chart, and was converted to the logarithm of the minimum angle of resolution (logMAR) VA. The change of VA (ΔVA, defined as end minus beginning) was calculated by comparing the VAs at the beginning and end of observation periods. Axial length (AL) was measured with optical biometry (OA-2000^®^; Tomey, Nagoya, Japan). ORA and CST measurements were carried out within 1 year from the end of the follow-up period. Diagnosis of secondary CNV in eyes with AS was made using funduscopic examinations and OCT scans. In all eyes, an intravitreal anti-VEGF injection with 30 gauge needle was carried out whenever secondary CNV was detected. A previous study indicated that corneal biomechanical properties don’t change with the minimal invasive vitreoretinal surgery (23 gauge vitrectomy)^11^, and hence the effect of this injection procedure on the corneal biomechanical properties would be negligible.

### Measurements in normal subjects

Following comprehensive ophthalmic examinations including the measurement of BCVA, AL, slit-lamp biomicroscopy, and funduscopy, ORA and CST measurements were carried out on the same day.

### Ocular Response Analyzer (ORA)

The details of the ORA measurement were described previously^12^. Briefly, the ORA records two applanation pressures, prior to and following an indentation of the cornea with the application of a rapid air jet. Due to its viscoelastic property, the cornea resists the air puff, resulting in a delay in the outward corneal movement, which causes the difference in the pressures at the inward and outward applanations. This difference is called CH^13^. The CRF is also calculated using the difference between the inward and outward pressure, but indicates the elastic property of the cornea^14^. ORA measurements were carried out three times with at least a 5-minute interval between each measurement, and the average value was used in the analysis. All data had a quality index of more than 7.5 as recommended by the manufacturer.

### Corvis ST (CST) tonometer

**Figure 1:**
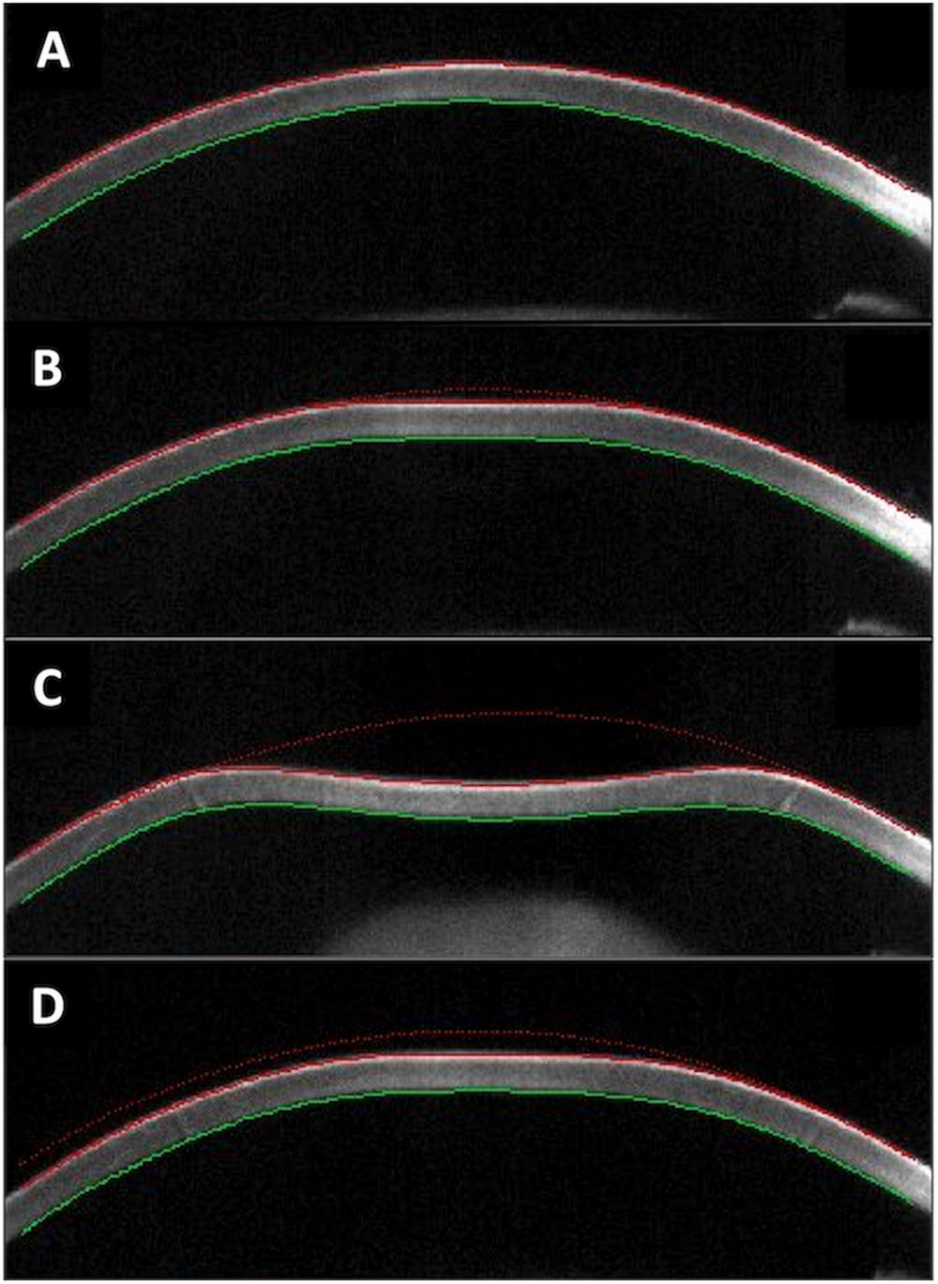
Corneal deformation during the Corvis ST measurement. Corvis ST tonometer emits a rapid air puff, and corneal deformation is recorded using a high-speed Scheimpflug camera. (**A**) shows an image prior to air puff applanation, (**B**) shows an image in the first applanation, (**C**) shows an image at the highest concavity, and (**D**) shows an image in the second applanation.

As detailed elsewhere^15^, CST, a non-contact tonometer, monitors the corneal response to an air puff pulse with a high-speed Scheimpflug camera, capturing 4,330 frames per second (Fig. 1)^12^. Corneal biomechanical characteristics are assessed by analyzing these images, and five main screening parameters including Deformation amplitude (DA) ratio, Integrated radius (IR), Ambrosio relational thickness horizontal (ARTh), and stiffness parameter at first applanation (SP-A1); Corvis biomechanical index (CBI) is calculated and displayed on the Vincigurra screening report with the current software version 1.3r1538^16^. The DA ratio describes the relationship between deformation amplitude at the apex and deformation amplitude at 2 mm. In a soft cornea, the corneal deformation starts at the center of the cornea, and the deformation of the pericentral cornea is limited, and as a result, the DA ratio value increases^17^. The ‘Integrated Radius’ is the area under the radius of the inversed curvature during the concave phase^10^. The ARTh represents the quotient of the corneal thickness at the thinnest point of the horizontal longitude and the thickness progression^10^. The SP-A1 is a stiffness parameter derived by dividing the loading amount by the amount of deformation^18^. The CBI is the combination of these parameters developed in an effort to detect subclinical keratoconus and corneal ectasia^10^. The central corneal thickness (CCT) was also measured with CST tonometry.

### Statistical Analyses

A linear mixed model, whereby patients were regarded as a random effect, was used to compare baseline characteristics of each group with the exception of sex ratio, which was evaluated using the chi-square test. Eight variables of CH, CRF, CCT, DA ratio, Integrated Radius, ARTh, SP-A1, and CBI were compared between the AS and normal groups, using the liner mixed model.

In the AS group, ten variables of age at the beginning of observation, AL, CH, CRF, CCT, DA ratio, Integrated Radius, ARTh, SP-A1, and CBI were compared between the groups of eyes received intravitreal anti-VEGF injection [Injection (+) group] and those without the treatment [Injection (−) group], using the linear mixed model.

The relationship between F_IV_ and the variables of initial age, AL, CCT, CH, CRF, DA ratio, Integrated Radius, ARTh, SP-A1, and CBI were also investigated using the liner mixed model. The model selection used to identify the optimal linear mixed model for F_IV_ was carried out using the second-order bias-corrected Akaike information criterion (AICc) index, from all 2^10^ patterns consisting of ten variables (initial age, AL, CH, CRF, CCT, DA ratio, Integrated Radius, ARTh, SP-A1, and CBI). The AIC is a well-known statistical measurement used in model selection, and the AICc is a corrected version of the AIC, which provides an accurate estimation even when the sample size is small^19^. The log-likelihood of a paired model was compared using the analysis of variance (ANOVA) test. The selected variables through the model selection were regarded as statistically significant. It is more fruitful to determine the relative importance to the contributions of, and interactions between, a number of processes^20^. The marginal R-squared (mR^2^) value was calculated following a method proposed by Nakagawa and Holger^21^.

The association between change in VA (ΔVA) and the same ten variables was also investigated using the linear mixed model, followed by the model selection with AICc.

All statistical analyses were performed using R (version 3.4.1, http://www.R-project.org/).

## Results

The AS group consisted of 28 eyes of 15 patients with AS and the control group included 30 eyes of 27 subjects. Table 1 summarizes the baseline characteristics of these groups; no significant difference was observed between the two groups.

**Table 1:** Baseline Characteristics of the Angioid Streaks and Control Groups.

	Angioid Streaks	Control	*P* value*
	N = 28	N = 30	
Age [years]	67.9 ± 9.8	73.6 ± 7.7	0.064
Sex, male (%)	13 (46.4%)	15 (50.0%)	0.99
LogMAR	0.45 ± 0.6	0.0022 ± 0.2	0.0006
AL [mm]	24.5 ± 1.6	23.7 ± 1.4	0.15

AL, Axial length; LogMAR, logarithm of the minimum angle of resolution. *Comparison between the Angioid Streaks and control group. The linear mixed modeling was used except for sex ratio, which was compared with the chi-square test.

Table 2 shows the comparisons of corneal biomechanical characteristics between the AS and normal groups. None of the corneal biomechanical characteristics were significantly different between these two groups (p > 0.05, linear mixed model), except for CCT, which was significantly thicker in the AS group (p = 0.048).

**Table 2:** Univariate Analyses of Corneal Biomechanical Properties in the Angioid Streaks and Control Groups.

Characteristics	Angioid Streaks	Control	*P* Value*
	N = 28	N = 30	
CH [mmHg]	10.1 ± 0.8	9.75 ± 0.8	0.16
CRF [mmHg]	10.1 ± 1.3	9.55 ± 1.3	0.20
CCT [μm]	554 ± 29	541 ± 21	0.048
DA ratio (2 mm)	4.14 ± 3.9	4.24 ± 0.34	0.34
IR [mm^−1^]	7.40 ± 1.1	7.61 ± 0.87	0.38
ARTh	518 ± 103	526 ± 155	0.96
SP-A1	107 ± 15	100 ± 16	0.23
CBI	0.094 ± 0.3	0.11 ± 0.2	0.72

CH, Corneal hysteresis; CRF, Corneal resistance factor; DA ratio, Deformation Amplitude ratio; IR, Integrated Radius; ARTh, Ambrosio Relational Thickness horizontal; SP-A1, Stiffness parameter at first applanation; CBI: Corvis Biomechanical Index; CCT, Central corneal thickness.

*Comparison between the Angioid Streaks and control groups (linear mixed model).

**Figure 2:**
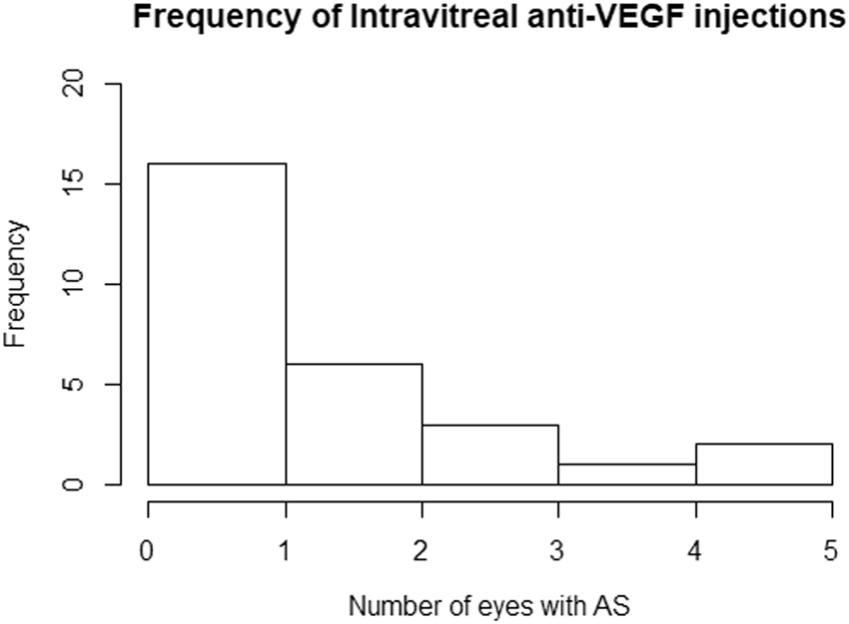
Histogram of the frequency of intravitreal anti-VEGF injections (per year) in the AS group. AS eyes had intravitreal anti-VEGF injections 6.1 ± 8.7 times in the observation period of 5.7 ± 2.5 years (1.1 ± 1.4 times per year). CNV never progressed in 12 eyes without the intravitreal anti-VEGF injection. VEGF, vascular endothelial growth factor; AS, angioid streaks; CNV, choroidal neovascularization.

**Figure 3:**
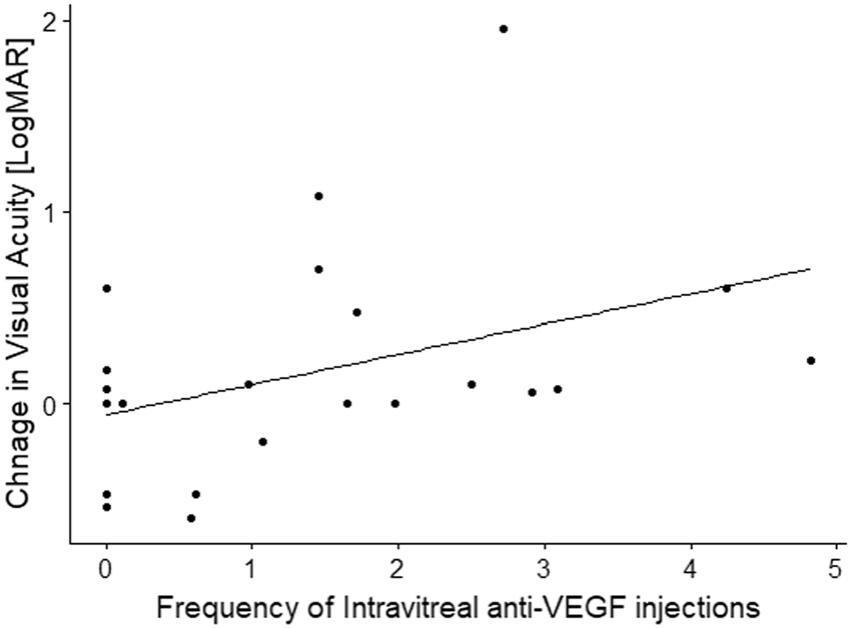
Relationship between change in visual acuity and numbers of intravitreal anti-VEGF injections in Angioid streaks patients. In the eyes with Angiod streaks, change in visual acuity was significantly associated with frequency of intravitreal anti-VEGF injection (coefficient = 0.16, standard error (SE) = 0.072, p = 0.043, linear mixed model).

The AS eyes had intravitreal anti-VEGF injections 6.1 ± 8.7 times in the observation period of 5.7 ± 2.5 years (1.1 ± 1.4 times per year). Figure 2 shows the histogram of F_IV_ in the AS group. In the AS eyes, ΔVA was significantly associated with F_IV_ [coefficient = 0.16, standard error (SE) = 0.072, p = 0.043, linear mixed model] (Fig. 3).

In the AS eyes, the optimal linear model identified for F_IV_ was; F_IV_ = −11.9–1.1 (SE = 0.35, p = 0.010) × CH + 1.4 (SE = 0.34, p = 0.0020) × CRF + 2.4 (SE = 0.91, p = 0.027) × DA Ratio (AICc = 98.2, mR^2^ = 0.40, Model 1).

This optimal model was significantly better than the model without the DA ratio (p = 0.0053, ANOVA), and was significantly better than the model without CH and CRF. (p = 0.0013, ANOVA).

In the AS eyes, 16 eyes were in the Injection (+) group and 12 eyes were in the Injection (−) group. Table 3 shows the results of comparisons of age at the beginning of observation, and the AL and the corneal biomechanical properties between the Injection (+) and Injection (−) groups. There was no statistical difference between the two groups in any parameters (p > 0.05, linear mixed model).

**Table 3:** Univariate Analysis of Corneal Biomechanical Properties in the Injection (+) and Injection (−) Eyes with Angioid Streaks (AS).

Characteristics	AS: Injection (+)	AS: Injection (−)	*P* Value*
	N = 16	N = 12	
Age at the beginning of observation	63.5 ± 8.1	60.2 ± 11	0.077
AL [mm]	24.5 ± 1.6	24.4 ± 1.6	0.42
CH [mmHg]	10.0 ± 0.8	10.1 ± 0.9	0.87
CRF [mmHg]	10.3 ± 1.1	9.93 ± 1.6	0.19
CCT [μm]	552 ± 32	558 ± 28	0.18
DA ratio (2 mm)	4.16 ± 0.35	4.12 ± 0.47	0.27
IR [mm^−1^]	7.30 ± 0.91	7.53 ± 1.3	0.37
ARTh	537 ± 111	492 ± 90	0.57
SP-A1	105 ± 17	110 ± 11	0.11
CBI	0.13 ± 0.3	0.05 ± 0.15	0.39

AL, Axial length; CH, Corneal hysteresis; CRF, Corneal resistance factor; DA ratio, Deformation Amplitude ratio; IR, Integrated Radius; ARTh, Ambrosio Relational Thickness horizontal; SP-A1, Stiffness parameter at first applanation; CBI, Corvis Biomechanical Index. *Comparison between Injection (+) eyes and Injection (−) eyes with Angioid Streaks (linear mixed model).

In the Injections (+) group, the optimal linear model for ΔVA was: ΔVA = −17.3–0.19 (SE = 0.19, p = 0.62) × CH + 1.2 (SE = 0.12, p = 0.50) × CRF + 2.7 (SE = 0.51, p = 0.32) × DA Ratio − 0.0079 (SE = 0.0010, p = 0.43) × ARTh (AICc = 63.6, mR^2^ = 0.55, Model 2).

## Discussion

In the current study, corneal biomechanical properties were measured using ORA and CST, in eyes with AS and normal eyes. No significant difference was observed in the corneal biomechanical properties between the AS and normal eyes, except for CCT. However, ORA-CH, ORA-CRF, and the CST-DA ratio were associated with the activity of AS, and was calculated as the frequency of intravitreal anti-VEGF injections. Moreover, ORA-CH, ORA-CRF, the CST-DA ratio, and the CST-ARTh were associated with the VA outcome in the AS eyes with intravitreal anti-VEGF injections.

The current study indicated that both CH and CRF, measured with ORA, were associated with the activity of AS, as suggested by F_IV_. More specifically, CH was negatively associated with F_IV_; lower CH was associated with the activity of AS. It has been reported that eyes were deformed even in daily life activities, such as postural change^22^, eye lid blinking^23^, ocular pulsatility due to ocular hemodynamics^24^, Valsalva maneuver^25^, and also eye movement was a stress to an eye^26^. An eye with high hysteresis is more likely to absorb these external strains with the damping capacity, which may be advantageous to prevent the occurrence of CNV. The mechanism of governing the hysteresis in the cornea is not entirely clear; however, it is likely that this is due to the corneal lamellae organization because collagen goes through a sequence of deformation steps beginning with a macroscopic uncrimping of collagen, followed by the removal of microscopic kinks in collagen molecules within the gaps of fibril, and finally sliding of collagen molecules^27–30^. This phenomenon takes place not only in the cornea, but also in the sclera^31,32^. Indeed, the cornea and sclera are continuous collagenous structures of an eye, and the biomechanical characteristics of the cornea and sclera may be similar^3^. Embryologically, the sclera and Bruch’s membrane are both derived from neural crest^33^, which implies that corneal biomechanical properties are associated with biomechanical properties of the Bruch’s membrane.

CRF is a parameter of the resistance of the cornea, and it has been reported that it is elevated in stiff corneas *in vivo*^3^. In the current study, CRF was positively associated with F_IV_, as suggested by Model 1. This may be because, in a rigid (high CRF) eye, an extrinsic force to the anterior segment of the eye is directly transferred to the posterior segment of the eye, because of the poor energy absorption (low hysteresis). This continuous mechanical stress at the posterior segment of an eye may be disadvantageous for the prevention of the development of CNV. The current study also showed that the CST-measured DA ratio was also related to F_IV_; a high DA ratio was associated with the increase of F_IV_. The DA ratio describes the relationship between corneal deformation amplitude at the apex and 2 mm, and DA ratio values increase in corneas with smaller coordinal movement^17^. This result also supported our finding that an AS eye with a rigid cornea required more frequent intravitreal anti-VEGF injections (active AS). Both ORA and CST measure corneal biomechanical properties; however, they measured different aspects^12^, because of the different measurement mechanisms. ORA measures the difference of pressure values at first and second applanation, whereas CST measures corneal morphological changes following the application of an air pulse. In the current study, it was suggested that it may be useful to analyze the activity of AS by measuring corneal biomechanical properties using both the ORA and CST methodologies.

In the current study, CRF was not significantly different between the AS and normal eyes. However, a previous study reported that CRF was significantly increased in eyes with AS compared to normal eyes^3^. These controversial results may be due to the differences of the study populations; the previous study was designed as an age- and sex- matched study, where much younger aged subjects (average, 48.2 years of age) were used than in the current study (average, 67.9 years of age). Considering that both ORA- and CST-measured parameters showed no significant difference between two groups, we believe our results were not misleading.

Interestingly, while all examined cornel biomechanical properties in AS eyes showed no significant difference compared to normal eyes, the poor prognosis of VA was associated with the high F_IV_, low CH, high CRF, and high DA ratio. This was probably because a change in the poor prognosis of VA was significantly related to increased F_IV_. A poor prognosis of VA was also associated with low ARTh, which reflects the asymmetric pachymetrical distribution of the cornea, such as in keratoconus eyes. In keratoconus eyes, the rigidity of the cornea is decreased and the CRF is low^34^.

In the current study, the CCT was thicker in the AS eyes than in the normal eyes. A previous study reported that there was no significant difference in CCT between AS and control eyes. However, there was no previous study investigating the anatomical structure of the cornea in AS eyes. It has been reported that the elastin microfibrillar bundles are significantly reduced in a mouse model of Marfan’s syndrome, which is a type of collagen disease^35^. The reason for our current results is not entirely clear, however, the corneal stromal structure might also be affected by structural changes in collagen in AS eyes, similar to the eyes with Marfan’s syndrome. Despite this significant differences, the CCT was not selected in the optimal model for F_IV_ (Model 1), suggesting that other corneal biomechanical properties better explained the activity of AS.

There are a few limitations in the current study. First, the parameters of ORA and CST were investigated from retrospectively collected F_IV_ data. This limitation may not have affected the results, because intravitreal anti-VEGF injection does not affect the anterior segment structures of eyes^36^, However, a further prospective study should be carried out to confirm the current results. Second, the eyes with refractive error more than plus/minus 2.0 diopter were excluded in the current study. In the current study, we attempted to elucidate the influence of the corneal biomechanical properties angioid streaks, excluding the effect of a myopic change, since corneal biomechanical properties are affected by myopic change. However, in the real world clinic, eyes with angioid streaks are often complicated with myopic change. Thus a further investigation would be needed shedding light on this issue. Third, the sample size was limited in the current study. A study with larger number of patients would be preferred to confirm the results obtained.

In conclusion, there was no significant difference in corneal biomechanical properties between AS and normal eyes, except for CCT. Corneal biomechanical properties were associated with the activity and VA outcome in AS.
